# Psychophysical Tests of the Hypothesis of a Bottom-Up Saliency Map in Primary Visual Cortex

**DOI:** 10.1371/journal.pcbi.0030062

**Published:** 2007-04-06

**Authors:** Li Zhaoping, Keith A May

**Affiliations:** Department of Psychology, University College London, London, United Kingdom; University College London, United Kingdom

## Abstract

A unique vertical bar among horizontal bars is salient and pops out perceptually. Physiological data have suggested that mechanisms in the primary visual cortex (V1) contribute to the high saliency of such a unique basic feature, but indicated little regarding whether V1 plays an essential or peripheral role in input-driven or bottom-up saliency. Meanwhile, a biologically based V1 model has suggested that V1 mechanisms can also explain bottom-up saliencies beyond the pop-out of basic features, such as the low saliency of a unique conjunction feature such as a red vertical bar among red horizontal and green vertical bars, under the hypothesis that the bottom-up saliency at any location is signaled by the activity of the most active cell responding to it regardless of the cell's preferred features such as color and orientation. The model can account for phenomena such as the difficulties in conjunction feature search, asymmetries in visual search, and how background irregularities affect ease of search. In this paper, we report nontrivial predictions from the V1 saliency hypothesis, and their psychophysical tests and confirmations. The prediction that most clearly distinguishes the V1 saliency hypothesis from other models is that task-irrelevant features could interfere in visual search or segmentation tasks which rely significantly on bottom-up saliency. For instance, irrelevant colors can interfere in an orientation-based task, and the presence of horizontal and vertical bars can impair performance in a task based on oblique bars. Furthermore, properties of the intracortical interactions and neural selectivities in V1 predict specific emergent phenomena associated with visual grouping. Our findings support the idea that a bottom-up saliency map can be at a lower visual area than traditionally expected, with implications for top-down selection mechanisms.

## Author Summary

Only a fraction of visual input can be selected for attentional scrutiny, often by focusing on a limited extent of the visual space. The selected location is often determined by the bottom-up visual inputs rather than the top-down intentions. For example, a red dot among green ones automatically attracts attention and is said to be salient. Physiological data have suggested that the primary visual cortex (V1) in the brain contributes to creating such bottom-up saliencies from visual inputs, but indicated little on whether V1 plays an essential or peripheral role in creating a saliency map of the input space to guide attention. Traditional psychological frameworks, based mainly on behavioral data, have implicated higher-level brain areas for the saliency map. Recently, it has been hypothesized that V1 creates this saliency map, such that the image location whose visual input evokes the highest response among all V1 output neurons is most likely selected from a visual scene for attentional processing. This paper derives nontrivial predictions from this hypothesis and presents their psychophysical tests and confirmations. Our findings suggest that bottom-up saliency is computed at a lower brain area than previously expected, and have implications on top-down attentional mechanisms.

## Introduction

Visual selection of inputs for detailed, attentive, processing often occurs in a bottom-up or stimulus driven manner, particularly in selections immediately or very soon after visual stimulus onset [1–3]. For instance, a vertical bar among horizontal ones or a red dot among green ones perceptually pops out automatically to attract attention [4,5], and is said to be highly salient pre-attentively. Physiologically, a neuron in the primary visual cortex (V1) gives a higher response to its preferred feature, e.g., a specific orientation, color, or motion direction, within its receptive field (RF) when this feature is unique within the display, rather than when it is one of the elements in a homogenous background [6–12]. This is the case even when the animal is under anesthesia [9], suggesting bottom-up mechanisms. This occurs because the neuron's response to its preferred feature is often suppressed when this stimulus is surrounded by stimuli of the same or similar features. Such contextual influences, termed iso-feature suppression, and iso-orientation suppression in particular, are mediated by intracortical connections between nearby V1 neurons [13–15]. The same mechanisms also make V1 cells respond more vigorously to an oriented bar when it is at the border, rather than at the middle, of a homogeneous orientation texture, as physiologically observed [10], since the bar has fewer iso-orientation neighbors at the border. These observations have prompted suggestions that V1 mechanisms contribute to bottom-up saliency for pop-out features like the unique orientation singleton or the bar at an orientation texture border (e.g., [6–10]). This is consistent with observations that highly salient inputs can bias responses in extrastriate areas receiving inputs from V1 [16,17].

Behavioral studies have examined bottom-up saliencies extensively in visual search and segmentation tasks [4,18,19], showing more complex, subtle, and general situations beyond basic feature pop-outs. For instance, a unique feature conjunction, e.g., a red vertical bar as a color-orientation conjunction, is typically less salient and requires longer search times; ease of searches can change with target-distractor swaps; and target salience decreases with background irregularities. However, few physiological recordings in V1 have used stimuli of comparable complexity, leaving it open how generally V1 mechanisms contribute to bottom-up saliency.

Meanwhile, a model of contextual influences in V1 [20–23], including iso-feature suppression and colinear facilitation [24,25], has demonstrated that V1 mechanisms can plausibly explain these complex behaviors mentioned above, assuming that the V1 cell with the highest response to a target determines its salience and thus the ease of a task. Accordingly, V1 has been proposed to create a bottom-up saliency map, such that the RF location of the most active V1 cell is most likely selected for further detailed processing [20,23]. We call this proposal the V1 saliency hypothesis. This hypothesis is consistent with the observation that microstimulation of a V1 cell can drive saccades, via superior colliculus, to the corresponding RF location [26], and that higher V1 responses correlate with shorter RTs to saccades to the corresponding RFs [27]. It can be clearly expressed algebraically. Let (*O*_1_,*O*_2_,…*O*_M_) denote outputs or responses from V1 output cells indexed by *i* = 1, 2,...*M*, and let the RFs of these cells cover locations (*x*_1_,*x*_2_,…*x_M_*), respectively, then the location selected by bottom-up mechanisms is *◯* = *x_î_* where *î* is the index of the most responsive V1 cell (mathematically, *î* = argmax*_i_O_i_*). It is then clear that (1) the saliency SMAP(*x*) at a visual location *x* increases with the response level of the most active V1 cell responding to it,

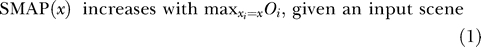
and the less-activated cells responding to the same location do not contribute, regardless of the feature preferences of the cells; and (2) the highest response to a particular location is compared with the highest responses to other locations to determine the saliency of this location, since only the RF location of the most activated V1 cell is the most likely selected (mathematically, the selected location is *◯* = argmax*_x_*SMAP(*x*))). As salience merely serves to order the priority of inputs to be selected for further processing, only the order of the salience is relevant [23]. However, for convenience we could write Equation 1 as SMAP(*x*) = [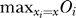 ] /[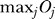 ], or simply SMAP(*x*) = 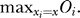 Note that the interpretation of *x_i_* = *x* is that the RF of cell *i* covers location *x* or is centered near *x*.

In a recent physiological experiment, Hegde and Felleman [28] used visual stimuli composed of colored and oriented bars resembling those used in experiments on visual search. In some stimuli the target popped out easily (e.g., the target had a different color or orientation from all the background elements), whereas in others, the target was more difficult to detect, and did not pop out (e.g., a color-orientation conjunction search, where the target is defined by a specific combination of orientation and color). They found that the responses of the V1 cells, which are tuned to both orientation and color to some degree, to the pop-out targets were not necessarily higher than responses to non-pop-out targets, and thus raising doubts regarding whether bottom-up saliency is generated in V1. However, these doubts do not disprove the V1 saliency hypothesis since the hypothesis does not predict that the responses to pop-out targets in some particular input images would be higher than the responses to non-pop-out targets in other input images. For a target to pop out, the response to the target should be substantially higher than the responses to all the background elements. The absolute level of the response to the target is irrelevant: what matters is the relative activations evoked by the target and background. Since Hegde and Felleman [28] did not measure the responses to the background elements, their findings do not tell us whether V1 activities contribute to saliency. It is likely that the responses to the background elements were higher for the conjunction search stimuli, because each background element differed greatly from many of its neighbors, and, as for the target, there would have been weak iso-feature suppression on neurons responding to the background elements. On the other hand, each background element in the pop-out stimuli always had at least one feature (color or orientation) the same as all of its neighbors, so iso-feature suppression would have reduced the responses to the background elements, making them substantially lower than the response to the target. Meanwhile, it remains difficult to test the V1 saliency hypothesis physiologically when the input stimuli are more complex than those of the singleton pop-out conditions.

Psychophysical experiments provide an alternative means to ascertain V1′s role in bottom-up salience. While previous works [20–23] have shown that the V1 mechanisms can plausibly explain the commonly known behavioral data on visual search and segmentation, it is important to generate from the V1 saliency hypothesis behavioral predictions that are hitherto unknown experimentally so as to test the hypothesis behaviorally. This hypothesis testing is very feasible for the following reasons. There are few free parameters in the V1 saliency hypothesis since (1) most of the relevant physiological mechanisms in V1 are established experimental facts that can be modeled but not arbitrarily distorted, and (2) the only theoretical input is the hypothesis that the RF location of the most responsive V1 cell to a scene is the most likely selected. Consequently, the predictions from this hypothesis can be made precise, making the hypothesis falsifiable. One such psychophysical test confirming a prediction has been reported recently [29]. The current work aims to test the hypothesis more systematically, by providing nontrivial predictions that are more indicative of the particular nature of the V1 saliency hypothesis and the V1 mechanisms.

For our purpose, we first review the relevant V1 mechanisms in the rest of the Introduction section. The Results section reports the derivations and tests of the predictions. The Discussion section will discuss related issues and implications of our findings, discuss possible alternative explanations for the data, and compare the V1 saliency hypothesis with traditional saliency models [18,19,30,31] that were motivated more by the behavioral data [4,5] than by their physiological basis.

The relevant V1 mechanisms for the saliency hypothesis are the RFs and contextual influences. Each V1 cell [32] responds only to a stimulus within its classical receptive field (CRF). Input at one location *x* evokes responses (*O_i_*,*O_j_*…) from multiple V1 cells *i, j,*… having overlapping RFs covering *x*. Each cell is tuned to one or more particular features including orientation, color, motion direction, size, and depth, and increases its response monotonically with the input strength and resemblance of the stimulus to its preferred feature. We call cells tuned to more than one feature dimension conjunctive cells [23]; e.g., a vertical rightward conjunctive cell is simultaneously tuned to rightward motion and vertical orientation [32], a red horizontal cell to red color and horizontal orientation [33]. Hence, for instance, a red vertical bar could evoke responses from a vertical tuned cell, a red tuned cell, a red vertical conjunctive cell, and another cell preferring orientation two degrees from vertical but having an orientation tuning width of 15°, etc. The V1 saliency hypothesis states that the saliency of a visual location is dictated by the response of the most active cell responding to it [20,23,34], 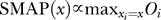 , rather than the sum of the responses 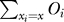 to this location. This makes the selection easy and fast, since it can be done by a single operation to find the most active V1 cell (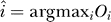 ) responding to any location and any feature(s). We will refer to saliency by the maximum response, 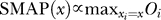 as the MAX rule, to saliency by the summed response 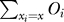 as the SUM rule. It will be clear later that the SUM rule is not supported, or is less supported by data, nor is it favored by computational considerations (see Discussion).

Meanwhile, intracortical interactions between neurons make a V1 cell's response context-dependent, a necessary condition for signaling saliency, since, e.g., a red item is salient in a green but not in a red context. The dominant contextual influence is the iso-feature suppression mentioned earlier, so that a cell responding to its preferred feature will be suppressed when there are surrounding inputs of the same or similar feature. Given that each input location will evoke responses from many V1 cells, and that responses are context-dependent, the highest response to each location to determine saliency will also be context-dependent. For example, the saliency of a red vertical bar could be signaled by the vertical tuned cell when it is surrounded by red horizontal bars, since the red tuned cell is suppressed through iso-color suppression by other red tuned cells responding to the context. However, when the context contains green vertical bars, its saliency will be signaled by the red tuned cells. In another context, the red vertical conjunctive cell could be signaling the saliency. This is natural since saliency is meant to be context-dependent.

Additional contextual influences, weaker than the iso-feature suppression, are also induced by the intracortical interactions in V1. One is the colinear facilitation to a cell's response to an optimally oriented bar when a contextual bar is aligned to this bar as if they are both segments of a smooth contour [24,25]. Hence, iso-orientation interaction, including both iso-orientation suppression and colinear facilitation, is not isotropic. Another contextual influence is the general, feature-unspecific, surround suppression to a cell's response by activities in nearby cells regardless of their feature preferences [6,7]. This causes reduced responses by contextual inputs of any features, and interactions between nearby V1 cells tuned to different features.

The most immediate and indicative prediction from the hypothesis is that task-irrelevant features can interfere in tasks that rely significantly on saliency. This is because at each location, only the response of the most activated V1 cell determines the saliency. In particular, if cells responding to task-irrelevant features dictate saliencies at some spatial locations, the task-relevant features become “invisible” for saliency at these locations. Consequently, visual attention is misled to task-irrelevant locations, causing delay in task completion. Second, different V1 processes for different feature dimensions are predicted to lead to asymmetric interactions between features for saliency. Third, the spatial or global phenomena often associated with visual grouping are predicted. This is because the intracortical interactions depend on the relative spatial relationship between input features, particularly in a non-isotropic manner for orientation features, making saliency sensitive to spatial configurations, in addition to the densities, of inputs. These broad categories of predictions will be elaborated in the next section in various specific predictions, together with their psychophysical tests.

## Results

For visual tasks in which saliency plays a dominant or significant role, the transform from visual input to behavioral response, particularly in terms of the RT in performing a task, via V1 and other neural mechanisms, can be simplistically and phenomenologically modeled as follows for clarity of presentation.

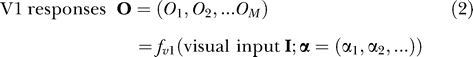


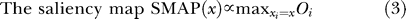


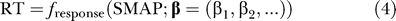
where *f*_*v*1_(.) models the transform from visual input **I** to V1 responses **O** via neural mechanisms parameterized by **α** describing V1′s RFs and intracortical interactions, while *f*_response_(.) models the transform from the saliency map SMAP to RT via the processes parameterized by ***β*** modeling decision making, motor responses, and other factors beyond bottom-up saliency. Without quantitative knowledge of ***β*** *,* it is sufficient for our purpose to assume a monotonic transform *f*_response_(.) that gives a shorter RT to a higher saliency value at the task-relevant location, since more salient locations are more quickly selected. This is of course assuming that the RT is dominated by the time for visual selection by saliency, or that the additional time taken after visual selection and before the task response, say indicated by button press, is a roughly constant quantity that does not vary sufficiently with the different stimuli being compared in any particular experiment. For our goal to test the saliency hypothesis, we will select stimuli such that this assumption is practically valid (see Discussion). Hence, all our predictions are qualitative; i.e., we predict a longer RT in one visual search task than that in another rather than the quantitative differences in these RTs. This does not mean that our predictions will be vague or inadequate for testing the V1 saliency hypothesis, since the predictions will be very precise by explicitly stating which tasks should require longer RTs than which other tasks, making them indicative of V1 mechanisms. Meanwhile, the qualitativeness makes the predictions robust and insensitive to variations in quantitative details parameterized by **α** of the underlying V1 mechanisms, such as the quantitative strengths of the lateral connections, provided that the qualitative facts of the V1 neural mechanisms are fixed or determined. Therefore, as will be clear below, our predictions can be derived and comprehensible merely from our qualitative knowledge of a few facts about V1; e.g., that neurons are tuned to their preferred features, that iso-feature suppression is the dominant form of contextual influences, that V1 cells tuned to color have larger RFs than cells tuned to orientation, etc, without resorting to quantitative model analysis or simulations which would only affect the quantitative but not the qualitative outcomes. Meanwhile, although one could quantitatively fit the model to behavioral RTs by tuning the parameters **α** and ***β*** (within the qualitative range), it adds no value since model fitting is typically possible given enough parameters, nor is it within the scope of this paper to construct a detailed simulation model that, for this purpose, would have to be more complex than the available V1 model for contextual influences [21–23]. Hence, we do not include quantitative model simulations in this study, which is only aimed at deriving and testing our qualitative predictions.

### Interference by Task-Irrelevant Features

Consider stimuli having two different features at each location, one task-relevant and the other task-irrelevant. For convenience, we call the V1 responses to the task-relevant and -irrelevant stimuli, relevant and irrelevant responses, respectively, and from the relevant and irrelevant neurons, respectively. If the irrelevant response(s) is stronger than the relevant response(s) at a particular location, this location's salience is dictated by the irrelevant response(s) according to the V1 saliency hypothesis, and the task-relevant features become “invisible” for saliency. In visual search and segmentation tasks that rely significantly on saliency to attract attention to the target or texture border, the task-irrelevant features are predicted to interfere with the task by directing attention irrelevantly or ineffectively.

Figure 1 shows the texture patterns (Figure 1A–1C) to illustrate this prediction. Pattern A has a salient border between two iso-orientation textures of left oblique and right oblique bars, respectively, activating two populations of neurons each for one of the two orientations. Pattern B is a uniform texture of alternating horizontal and vertical bars, evoking responses from another two groups of neurons for horizontal and vertical orientations, respectively. When all bars are of the same contrast, the neural response from the corresponding neurons to each bar would be the same (ignoring neural noise) if there were no intracortical interactions giving rise to contextual influences. With iso-orientation suppression, neurons responding to the texture border bars in pattern A are more active than neurons responding to other bars in pattern A; this is because they receive iso-orientation suppression from fewer active neighboring neurons, since there are fewer neighboring bars of the same orientation. For ease of explanation, let us say the highest neural responses to a border bar and a background bar are ten and five spikes/second, respectively. This V1 response pattern makes the border more salient, so it pops out in a texture-segmentation task. Each bar in pattern B has the same number of iso-orientation neighbors as a texture border bar in pattern A, so it evokes a comparable level of (highest) V1 response, i.e., ten spikes/second, to that evoked by a border bar in pattern A. If patterns A and B are superimposed, to give pattern C, the composite pattern will activate all neurons responding to patterns A and B, each neuron responding approximately as it does to A or B alone (for simplicity, we omitted the general suppression between neurons tuned to different orientations, without changing our conclusion, see below). According to the V1 saliency hypothesis, the saliency at each texture element location is dictated by the most activated neuron there. Since the (relevant) response to each element of pattern A is lower than or equal to the (irrelevant) response to the corresponding element of pattern B, the saliency at each element location in pattern C is the same as for B, so there is no texture border highlight in such a composite stimulus, making texture segmentation difficult.

**Figure 1:**
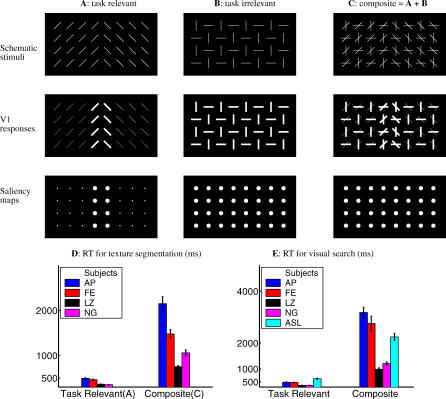
Prediction of Interference by Task-Irrelevant Features, and Its Psychophysical Test (A–C) Schematics of texture stimuli (extending continuously in all directions beyond the portions shown), each followed by schematic illustrations of its V1 responses, in which the orientation and thickness of a bar denote the preferred orientation and response level, respectively, of the activated neuron. Each V1 response pattern is followed below by a saliency map, in which the size of a disk, denoting saliency, corresponds to the response of the most activated neuron at the texture element location. The orientation contrasts at the texture border in (A) and everywhere in (B) lead to less suppressed responses to the stimulus bars since these bars have fewer iso-orientation neighbours to evoke iso-orientation suppression. The composite stimulus (C), made by superposing (A) and (B), is predicted to be difficult to segment, since the task-irrelevant features from (B) interfere with the task-relevant features from (A), giving no saliency highlights to the texture border. (D,E) RTs (differently colored data points denote different subjects) for texture segmentation and visual search tasks testing the prediction. For each subject, RT for the composite condition is significantly higher (*p* < 0.001). In all experiments in this paper, stimuli consist of 22 rows × 30 columns of items (of single or double bars) on a regular grid with unit distance 1.6° of visual angle.

For simplicity in our explanation, our analysis above included only the dominant form of contextual influence, the iso-feature suppression, but not the less dominant form of the contextual influence, the general surround suppression and colinear facilitation. Including the weaker forms of contextual influences, as in the real V1 or our model simulations [21–23], does not change our prediction here. So, for instance, general surround suppression between local neurons tuned to different orientations should reduce each neuron's response to pattern C from that to pattern A or B alone. Hence, the (highest) responses to the task-relevant bars in pattern C may be, say, eight and four spikes/second, respectively, at the border and background. Meanwhile, the responses to the task-irrelevant bars in pattern C should be, say, roughly eight spikes/second everywhere, leading to the same prediction of interference. In the rest of this paper, for ease of explanation without loss of generality or change of conclusions, we include only the dominant iso-feature suppression in our description of the contextual influences, and ignore the weaker or less dominant colinear facilitation and general surround suppression unless their inclusion makes a qualitative or relevant difference (as we will see in the section Emergent Grouping of Orientation Features by Spatial Configurations). For the same reason, our arguments do not detail the much weaker responses from cells not as responsive to the stimuli concerned, such as responses from motion direction selective cells to a nonmoving stimulus, or the response from a cell tuned to 22.5° to a texture element in pattern C composed of two intersecting bars oriented at 0° and 45°, respectively. (Jointly, the two bars resemble a single bar oriented at 22.5° only at a scale much larger or coarser than their own. Thus, the most activated cell tuned to 22.5° would have a larger RF, much of which would contain no (contrast or luminance) stimulus, leading to a response weaker than cells preferring both the scale and the orientation of the individual bars.) This is because these additional but nondominant responses at each location are “invisible” to saliency by the V1 saliency hypothesis and thus do not affect our conclusions.

Figure 1D shows that segmenting the composite texture C indeed takes much longer than segmenting the task-relevant component texture A, confirming the prediction. The RTs were taken in a task when subjects had to report the location of the texture border, as to the left or right of display center, as quickly as possible. (The actual stimuli used are larger, see Materials and Methods.) In pattern C, the task-irrelevant horizontal and vertical features from component pattern B interfere with segmentation by relevant orientations from pattern A. Since pattern B has spatially uniform saliency values, the interference is not due to the noisy saliencies of the background [19,35].

One may wonder whether each composite texture element in Figure 1C may be perceived by its average orientation at each location, see Figure 2F, thereby making the relevant orientation feature noisy to impair performance. Figure 2E demonstrates by our control experiment that this would not have caused as much impairment; RT for this stimulus is at least 37% shorter than that for the composite stimulus.

**Figure 2:**
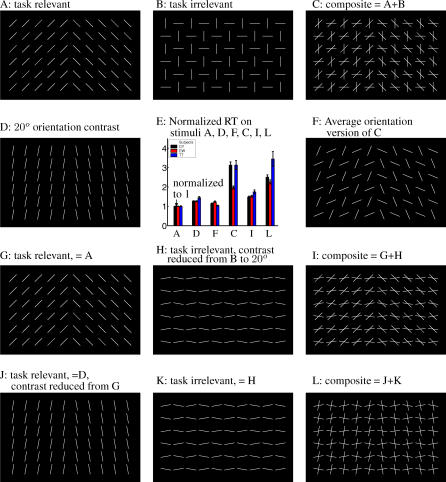
Further Illustrations To Understand Interference by Task-Irrelevant Features (A–C) As in Figure 1, the schematics of texture stimuli of various feature contrasts in task-relevant and -irrelevant features. (D) Like (A), except that each bar is 10° from vertical, reducing orientation contrast to 20°. (F) Derived from (C) by replacing each texture element of two intersecting bars by one bar whose orientation is the average of the original two intersecting bars. (G–I) Derived from (A–C) by reducing the orientation contrast (to 20°) in the interfering bars, each is 10° from horizontal. (J–L) Derived from (G–I) by reducing the task-relevant contrast to 20°. (E) Plots the normalized RTs for three subjects, DY, EW, and TT, on stimuli (A,D,F,C,I,L) randomly interleaved within a session. Each normalized RT is obtained by dividing the actual RT by the RT (which are 471, 490, and 528 ms, respectively, for subjects DY, EW, and TT) of the same subject for stimulus (A). For each subject, RT for (C) is significantly (*p* < 0.001) higher than that for (A,D,F,I) by at least 95%, 56%, 59%, and 29%, respectively. Matched sample *t*-test across subjects shows no significant difference (*p* = 0.99) between RTs for stimuli (C) and (L).

If one makes the visual search analog of the texture segmentation tasks in Figure 1, by changing stimulus Figure 1A (and consequently stimulus Figure 1C) such that only one target of left- (or right-) tilted bar is in a background of right- (or left-) tilted bars, qualitatively the same result (Figure 1E) is obtained. Note that the visual search task may be viewed as the extreme case of the texture-segmentation task when one texture region has only one texture element.

Note that, if saliency were computed by the SUM rule 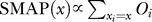 (rather than the MAX rule) to sum the responses *O_i_* from cells preferring different orientations at a visual location *x,* interference would not be predicted since the summed responses at the border would be greater than those in the background, preserving the border highlight. Here, the texture border highlight *H_border_* (for visual selection) is measured by the difference *H_border_* = *R_border_* − *R_ground_* between the (summed or maxed) response *R_border_* to the texture border and the response *R_ground_* to the background (where response *R_x_* at location *x* means 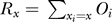 or 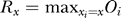 , under the SUM or MAX rule, respectively). This is justified by the assumption that the visual selection is by the winner-take-all of the responses *R_x_* in visual space *x,* hence the priority of selecting the texture border is measured by how much this response difference is compared with the level of noises in the responses. Consequently, the SUM rule applied to our example of response values gives the same border highlight *H_border_* = 5 spikes/second with or without the task-irrelevant bars, while the MAX rule gives *H_border_* = 0 and 5 spikes/second, respectively. If the border highlight is measured more conservatively by the ratio *H_border_* = *R_border_/R_ground_* (when a ratio *H_border_* = 1 means no border highlight), then the SUM rule predicts, in our particular example, *H_border_* = (10 + 10)/(5 + 10) = 4/3 with the irrelevant bars, and *H_border_* = 10/5 = 2 without, and thus some degree of interference. However, we argue below that even this measure of *H_border_* by the response ratio makes the SUM rule less plausible. Behavioral and physiological data suggest that, as long as the saliency highlight is above the just-noticable difference (JND, [36]), a reduction in *H_border_* should not increase RT as dramatically as observed in our data. In particular, previous findings [36,37] and our data (in Figure 2E) suggest that the ease of detecting an orientation contrast (assessed using RT) does not reduce by more than a small fraction when the orientation contrast is reduced, say, from 90° to 20° as in Figure 2A and Figure 2D [36,37], even though physiological V1 responses [38] to these orientation contrasts suggest that a 90° orientation contrast would give a highlight of *H*_90°_ ∼ 2.25 and a 20° contrast would give *H*_20°_ ∼ 1.25 using the ratio measurement for highlights. (Jones et al. [38] illustrated that the V1 response to a 90° and 20° orientation contrast, respectively, can be 45 and 25 spikes/second, respectively, over a background response of 20 spikes/second.) Hence, the very long RT in our texture segmentation with interference implies that the border should have a highlight *H_border_* ≈ 1 or below the JND, while a very easy segmentation without interference implies that the border should have *H_border_* ≫ 1. If *O_border_* and *O_ground_* are the relevant responses to the border and background bars, respectively, for our stimulus, and since *O_border_* also approximates the irrelevant response, then applying the SUM rule gives border highlight *H_border_* = 2*O_border_*/(*O_border_* + *O_ground_*) and *O_border_*/*O_ground_,* with and without interference, respectively. Our RT data thus require that *O_border_*/*O_ground_* ≫ 1 and 2*O_border_*/(*O_border_* + *O_ground_*) ≈ 1 should be satisfied simultaneously—this is difficult since *O_border_*/*_Oground_* > 2 means 2*O_border_*/(*O_border_* + *O_ground_*) > 4/3, and a larger *O_border_*/*O_ground_* would give a larger 2*O_border_*/(*O_border_* + *O_ground_*), making the SUM rule less plausible. Meanwhile, the MAX rule gives a border highlight *H_border_* = *O_border_*/*O_border_* = 1 with interference and *H_border_* = *O_border_*/*O_ground_* > 1 without. These observations strongly favor the MAX over the SUM rule, and we will show more data to differentiate the two rules later.

From our analysis above, we can see that the V1 saliency hypothesis also predicts a decrease of the interference if the irrelevant feature contrast is reduced, as demonstrated when comparing Figure 2G–2I with Figure 2A–2C, and confirmed in our data (Figure 2E). The neighboring irrelevant bars in Figure 2I are more similarly oriented, inducing stronger iso-feature suppression between them, and decreasing their evoked responses, say, from ten to seven spikes/second. (Although colinear facilitation is increased by this stimulus change, since iso-orientation suppression dominates colinear facilitation physiologically, the net effect is decreased responses to all the task-irrelevant bars.) Consequently, the relevant texture border highlights are no longer submerged by the irrelevant responses. The degree of interference would be much weaker, though still nonzero, since the irrelevant responses (of seven spikes/second) still dominate the relevant responses (of five spikes/second) in the background, reducing the relative degree of border highlight from five to three spikes/second. Analogously, interference can be increased by decreasing task-relevant contrast, as demonstrated by comparing Figure 2J–2L and Figure 2G–2I, and confirmed in our data (Figure 2E). Reducing the relevant contrast makes the relevant responses to the texture border weaker, say from ten to seven spikes/second, making these responses more vulnerable to being submerged by the irrelevant responses. Consequently, interference is stronger in Figure 2L than in Figure 2I. Essentially, the existence and strength of the interference depend on the relative response levels to the task-relevant and -irrelevant features, and these response levels depend on the corresponding feature contrasts and direct input strengths. When the relevant responses dictate saliency everywhere and their response values or overall response pattern are little affected by the existence or absence of the irrelevant stimuli, there should be little interference. Conversely, when the irrelevant responses dictate saliency everywhere, interference for visual selection is strongest. When the relevant responses dictate the saliency value at the location of the texture border or visual search target but not in the background of our stimuli, the degree of interference is intermediate. In both Figure 2C and Figure 2L, the irrelevant responses (approximately) dictate the saliency everywhere, so the texture borders are predicted to be equally nonsalient. This is confirmed across subjects in our data (Figure 2E), although there is a large variation between subjects, perhaps because the bottom-up saliency is so weak in these two stimuli that subject specific top-down factors contribute significantly to the RTs.

### The Color-Orientation Asymmetry in Interference

Can task-irrelevant features from another feature dimension interfere? Figure 3A illustrates orientation segmentation with irrelevant color contrasts. As in Figure 1, the irrelevant color contrast increases the responses to the color features since the iso-color suppression is reduced. At each location, the response to color could then compete with the response to the relevant orientation feature to dictate the saliency. In Figure 1C, the task-irrelevant features interfere because they evoke higher responses than the relevant features, as made clear by demonstrations in Figure 2. Hence, whether color can interfere with orientation or vice versa depends on the relative levels of V1 responses to these two feature types. Color and orientation are processed differently by V1 in two aspects. First, cells tuned to color, more than cells tuned to orientation, are usually in V1′s cytochrome oxidase–stained blobs which are associated with higher metabolic and neural activities [39]. Second, cells tuned to color have larger RFs [33,40]; hence, they are activated more by larger patches of color. In contrast, larger texture patches of oriented bars can activate more orientation-tuned cells, but do not make individual orientation-tuned cells more active. Meanwhile, in the stimulus for color segmentation (e.g., Figure 3B), each color texture region is large so that color-tuned cells are most effectively activated, making their responses easily the dominant ones. Consequently, the V1 saliency hypothesis predicts: (1) task-irrelevant colors are more likely to interfere with orientation than the reverse; (2) irrelevant color contrast from larger color patches can disrupt an orientation-based task more effectively than that from smaller color patches; and (3) the degree of interference by irrelevant orientation in color-based task will not vary with the patch size of the orientation texture.

**Figure 3:**
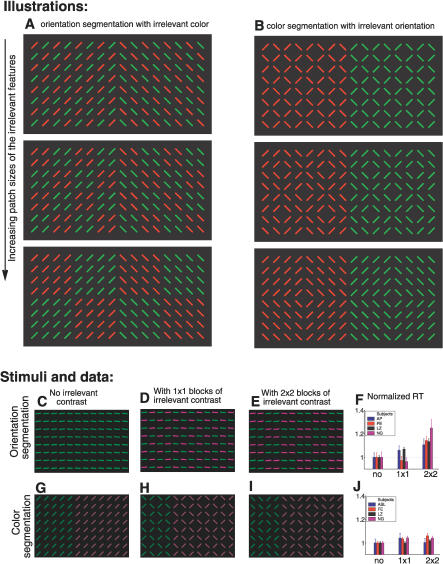
Interference between Orientation and Color, with Schematic Illustrations (Top [A,B]), and Stimuli/Data (Bottom [C–J]) (A) Orientation segmentation with irrelevant color. (B) Color segmentation with irrelevant orientation. (A,B) Larger patch sizes of irrelevant color gives stronger interference, but larger patch sizes of irrelevant orientation do not make interference stronger. (C–E) Small portions of the actual experimental stimuli for orientation segmentation, without color contrast (C) or with irrelevant color contrast in 1 × 1 (D) or 2 × 2 (E) blocks. All bars had color saturation *s_uv_* = 1, and were ±5° from horizontal. (F) Normalized RTs for (C–E) for four subjects (different colors indicate different subjects). The “no”, “1 × 1”, and “2 × 2” on the horizontal axis mark stimulus conditions for (C–E), i.e., with no or n × n blocks of irrelevant features. The RT for condition “2 × 2” is significantly longer (*p* < 0.05) than that for “no” in all subjects, and than that of “1 × 1” in three out of four subjects. By matched sample *t*-test across subjects, mean RTs are significantly longer in “2 × 2” than that in “no” (*p* = 0.008) and than that in “1 × 1” (*p* = 0.042). Each RT is normalized by dividing by the subject's mean RT for the “no” condition, which for the four subjects (AP, FE, LZ, NG) are 1170, 975, 539, and 1107 ms, respectively. (G–J) Color segmentation, analogous to (C–F), with stimulus bars oriented ±45° and of color saturation *s_uv_* = 0.5. Matched sample *t*-test across subjects showed no significant difference between RTs in different conditions. Only two out of four subjects had their RT significantly higher (*p* < 0.05) in interfering than in no interfering conditions. The un-normalized mean RTs of the four subjects (ASL, FE, LZ, NG) in “no” condition are: 650, 432, 430, 446 ms, respectively.

These predictions are apparent when viewing Figure 3A and 3B. They are confirmed by RT data for our texture segmentation task, shown in Figure 3C–3J. Irrelevant color contrast can indeed raise RT in orientation segmentation, but is effective only for sufficiently large color patches. In contrast, irrelevant orientation contrast does not increase RT in color segmentation regardless of the sizes of the orientation patches. In Figure 3C–3E, the irrelevant color patches are small, activating the color-tuned cells less effectively. However, interference occurs under small orientation contrast which reduces responses to relevant features (as demonstrated in Figure 2). Larger color patches can enable interference even to a 90° orientation contrast at the texture border, as apparent in Figure 3A, and has been observed by Snowden [41]. In Snowden's design, the texture bars were randomly rather than regularly assigned one of two iso-luminant, task-irrelevant colors, giving randomly small and larger sizes of the color patches. The larger color patches made task-irrelevant locations salient to interfere with the orientation segmentation task. Previously, the V1 saliency hypothesis predicted that Snowden's interference should become stronger when there are more irrelevant color categories; e.g., each bar could assume one of three rather than two different colors. This is because more color categories further reduce the number of iso-color neighbors for each colored bar and thus the iso-color suppression, increasing responses to irrelevant color. This prediction was subsequently confirmed [29].

In Figure 3G–3I, the relevant color contrast was made small to facilitate interference by irrelevant orientation, though unsuccessfully. Our additional data showed that orientation does not significantly interfere with color-based segmentation even when the color contrast was reduced further. The patch sizes, of 1 × 1 and 2 × 2, of the irrelevant orientation textures ensure that each bar in these patches evoke the same levels of responses, since each has the same number of iso-orientation neighbours (this would not hold when the patch size is 3 × 3 or larger). Such an irrelevant stimulus pattern evokes a spatially uniform level of irrelevant responses, thus ensuring that interference cannot possibly arise from non-uniform or noisy response levels to the background [19,35]. Patch sizes for irrelevant colors in Figure 3C–3E were made to match those of irrelevant orientations in Figure 3G–3I, so as to compare saliency effects by color and orientation features. Note that, as discussed in the section Interference by Task-Irrelevant Features, the SUM rule would predict the same interference only if saliency highlight *H_border_* is measured by the ratio between responses to the border and background. With this measure of *H_border_*, our data in this subsection, showing that the interference only increases RT by a small fraction, cannot sufficiently differentiate the MAX from the SUM rule.

### Advantage for Color-Orientation Double Feature but Not Orientation–Orientation Double Feature

A visual location can be salient due to two simultaneous feature contrasts. For instance, at the texture border between a texture of green, right-tilted bars and another texture of pink, left-tilted bars, in Figure 4C, both the color and orientation contrast could make the border salient. We say that the texture border has a color-orientation double-feature contrast. Analogously, a texture border of an orientation–orientation double contrast, and the corresponding borders of single-orientation contrasts, can be made as in Figure 4E–4G. We can ask whether the saliency of a texture border with a double-feature contrast can be higher than both of those of the corresponding single-feature–contrast texture borders. We show below that the V1 saliency hypothesis predicts a likely “yes” for color-orientation double feature but a definite “no” for orientation–orientation double feature.

**Figure 4:**
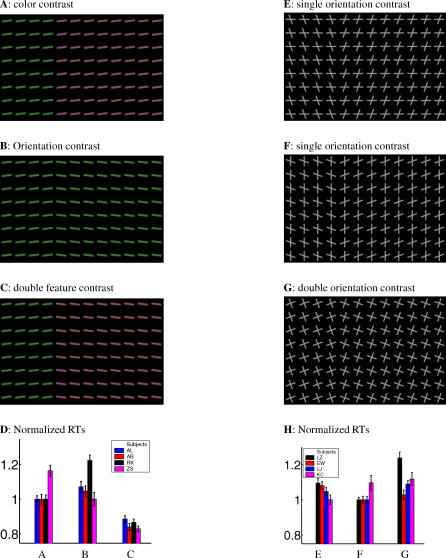
Small Portions of Actual Stimuli and Data in the Test of the Predictions of Saliency Advantage in Color-Orientation Double Feature (Left, [A–D]) and the Lack of It in Orientation–Orientation Double Feature (Right [E–H]) (A–C) Texture segmentation stimuli by color contrast, or orientation contrast, or by double color–orientation contrast. (D) Normalized RTs for the stimulus conditions (A–C). Normalization for each subject is by whichever is the shorter mean RT (which for the subjects AL, AB, RK, and ZS are, respectively, 651, 888, 821, and 634) of the two single-feature contrast conditions. All stimulus bars had color saturation *s_uv_ =* 0.2, and were *±*7.5° from horizontal. All subjects had their RT for the double-feature condition significantly shorter (*p* < 0.001) than those of both single-feature conditions. (E–G) Texture-segmentation stimuli by single- or double-orientation contrast, each oblique bar is *±*20° from vertical in (E) and *±*20° from horizontal in (F), and (G) is made by superposing the task-relevant bars in (E) and (F). (H) Normalized RTs for the stimulus conditions (E–G) (analogous to [D]). The shorter mean RT among the two single-feature conditions are, for four subjects (LZ, EW, LJ, KC), 493, 688, 549, 998 ms, respectively. None of the subjects had RT for (G) lower than the minimum of the RT for (E) and (F). Averaged over the subjects, the mean normalized RT for the double-orientation feature in (G) is significantly longer (*p* < 0.01) than that for the color-orientation double feature in (C).

V1 has color-orientation conjunctive cells that are tuned to both color and orientation, though their tuning to either feature is typically not as sharp as that of the single feature–tuned cells [33]. Hence, a colored bar can activate a color-tuned cell, an orientation-tuned cell, and a color-orientation conjunctive cell, with cell outputs *O_c_, O_o_,* and *O_co_*, respectively. The highest response max(*O_c_*,*O_o_*,*O_co_*) from these cells should dictate the saliency of the bar's location. Let the triplet of response be 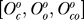 at an orientation texture border, 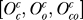 at a color border, and 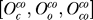 at a color-orientation double-feature border. Due to iso-feature suppression, responses of a single feature cell is higher with than without its feature contrast, i.e., 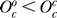 and 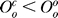 . The single-feature cells also have comparable responses with or without feature contrasts in other dimensions, i.e., 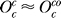 and 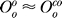 . Meanwhile, the conjunctive cell should have a higher response at a double than a single feature border, i.e., 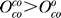 and 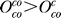 , since it has fewer neighboring conjunctive cells responding to the same color and same orientation. The maximum 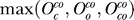 could be 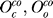 , or 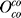 to dictate the saliency of the double-feature border. Without detailed knowledge, we expect that it is likely that, in at least some nonzero percentage of many trials, 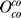 is the dictating response, and when this happens, 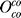 is larger than all responses from all cells to both single-feature contrasts. Consequently, averaged over trials, the double-feature border is likely more salient than both of the single-feature borders and thus should require a shorter RT to detect. In contrast, there are no V1 cells tuned conjunctively to two different orientations; hence, a double orientation–orientation border definitely cannot be more salient than both of the two single-orientation borders.

The above considerations have omitted the general suppression between cells tuned to different features. When this is taken into account, the single feature–tuned cells should respond less vigorously to a double feature than to the corresponding effective single feature contrast. This means, for instance, 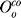 ≲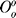 and 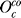 ≲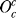 . This is because general suppression grows with the overall level of local neural activities. This level is higher with double-feature stimuli which activate some neurons more, e.g., when 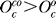 and 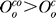 (at the texture border). In the color-orientation double-feature case, 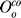 ≲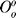 and 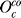 ≲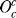 mean that 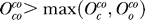 could not guarantee that 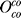 must be larger than all neural responses to both of the single feature borders. This consideration could somewhat weaken or compromise the double-feature advantage for the color-orientation case, and should make the double-orientation contrast less salient than the more salient one of the two single-orientation contrast conditions. In any case, the double-feature advantage in the color-orientation condition should be stronger than that of the orientation–orientation condition.

These predictions are indeed confirmed in the RT data. As shown in Figure 4D and 4H, the RT to locate a color-orientation double-contrast border Figure 4C is shorter than both RTs to locate the two single-feature borders Figure 4A and Figure 4B. Meanwhile, the RT to locate a double-orientation contrast of Figure 4G is no shorter than the shorter one of the two RTs to locate the two single-orientation contrast borders Figure 4E and Figure 4F. The same conclusion is reached (unpublished data) if the irrelevant bars in Figure 4E or Figure 4F, respectively, have the same orientation as one of the relevant bars in Figure 4F or Figure 4E, respectively. Note that, to manifest the double feature advantage, the RTs for the single-feature tasks should not be too short, since RT cannot be shorter than a certain limit for each subject. To avoid this RT floor effect, we have chosen sufficiently small feature contrasts to make RTs for the single-feature conditions longer than 450 ms for experienced subjects and even longer for inexperienced subjects.

Nothdurft [42] also showed the saliency advantage of the double-feature contrast in color orientation. The shortening of RT by feature doubling can be viewed phenomenologically as a violation of a race model which models the task's RT as the outcome of a race between two response decision making processes by color and orientation features, respectively. This violation has been used to account for the double-feature advantage in RT also observed in visual search tasks when the search target differs in both color and orientation from uniform distractors observed previously [43], and in our own data (Table 1A). In our framework, we could interpret the RT for color-orientation double feature as a result from a race between three neural groups—the color-tuned, the orientation-tuned, and the conjunctive cells.

**Table 1:**
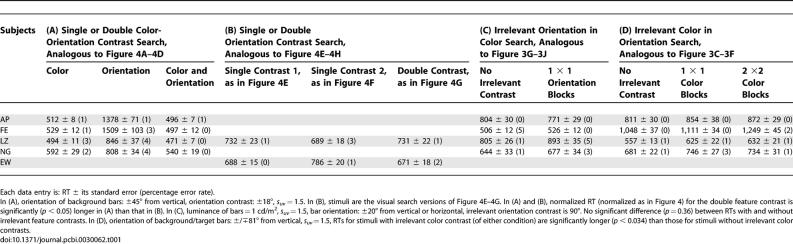
RTs (ms) in Visual Search for Unique Color and/or Orientation, Corresponding to Those in Figures 3 and 4

It is notable that the findings in Figure 4H cannot be predicted from the SUM rule. With single- or double-orientation contrast, the (summed) responses to the background bars are approximately unchanged, since the iso-orientation suppression between various bars is roughly unchanged. Meanwhile, the total (summed) response to the border is larger when the border has double-orientation contrast (even considering the general, feature unspecific, suppression between neurons). Hence, the SUM rule would predict that the double-orientation contrast border is more salient than the single-contrast one, regardless of whether one measures the border highlight *H_border_* by the difference or ratio between the summed response to the texture border and that to the background.

### Emergent Grouping of Orientation Features by Spatial Configurations

Combining iso-orientation suppression and colinear facilitation, contextual influences between oriented bars depend non-isotropically on spatial relationships between the bars. Thus, spatial configurations of the bars can influence saliency in ways that cannot be simply determined by densities of the bars, and properties often associated with grouping can emerge. Patterns A–G in Figure 5A–5G are examples of these, and the RT to segment each texture will be denoted as RT_A_, RT_B_, … , RT_G_. Patterns A and B both have a 90° orientation contrast between two orientation textures. However, the texture border in B seems more salient. Patterns C and D are both made by adding, to A and B, respectively, task-irrelevant bars ±45° relative to the task-relevant bars and containing a 90° irrelevant orientation contrast. However, the interference is stronger in C than in D. Patterns E and G differ from C by having zero orientation contrast among the irrelevant bars, pattern F differs from D analogously. As demonstrated in Figure 2, the interference in E and G should thus be much weaker than that in C, and that in F much weaker than that in D. The irrelevant bars are horizontal in E and vertical in G, on the same original pattern A containing only the ±45° oblique bars. Nevertheless, segmentation seems easier in E than in G. These peculiar observations all seem to relate to what is often called visual “grouping” of elements by their spatial configurations, and can in fact be predicted from the V1 saliency hypothesis when considering that the contextual influences between oriented bars are non-isotropic. To see this, we need to abandon the simplification used so far to approximate contextual influences by only the dominant component—iso-feature suppression. Specifically, we now include in the contextual influences the subtler components: (1) facilitation between neurons responding to colinear neighboring bars and (2) general feature-unspecific surround suppression between nearby neurons tuned to any features.

**Figure 5:**
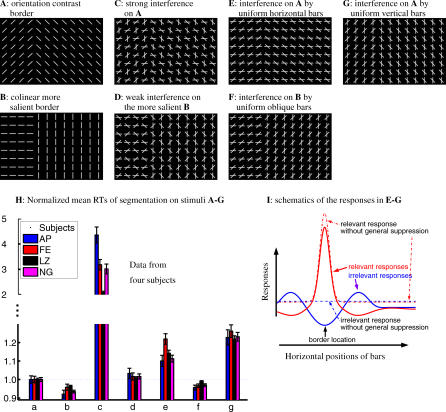
Demonstration and Testing the Predictions on Spatial Grouping (A–G) Portions of different stimulus patterns used in the segmentation experiments. Each row starts with an original stimulus (left) without task-irrelevant bars, followed by stimuli when various task-irrelevant bars are superposed on the original. (H) RT data when different stimulus conditions are randomly interleaved in experimental sessions. The un-normalized mean RT for four subjects (AP, FE, LZ, NG) in condition (A) are: 493, 465, 363, 351 ms. For each subject, it is statistically significant that RT_C_ > RT_A_ (*p* < 0.0005), RT_D_ > RT_B_ (*p* < 0.02), RT_A_ > RT_B_ (*p* < 0.05), RT_A_ < RT_E_, RT_G_ (*p* < 0.0005), RT_D_ > RT_F_, RT_C_ > RT_E_, RT_G_ (*p* < 0.02). In three out of four subjects, RT_E_ < RT_G_ (*p* < 0.01), and in two out of four subjects, RT_B_ < RT_F_ (*p* < 0.0005). Meanwhile, by matched sample *t*-tests across subjects, the mean RT values between any two conditions are significantly different (*p* smaller than values ranging from 0.0001 to 0.04). (I) Schematics of responses from relevant (red) and irrelevant (blue) neurons, with (solid curves) and without (dot-dashed curves) considering general suppressions, for situations in (E–G). Interference from the irrelevant features arises from the spatial peaks in their responses away from the texture border.

Due to colinear facilitation, a vertical border bar in pattern B is salient not only because a neuron responding to it experiences weaker iso-orientation suppression, but also because it additionally enjoys full colinear facilitation due to the colinear contextual bars, whereas a horizontal border bar in B, or an oblique border bar in A, has only half as many colinear neighbors. Hence, in an orientation texture, the vertical border bars in B, and in general colinear border bars parallel to a texture border, are more salient than border bars not parallel to the border given the same orientation contrast at the border. Hence, if the highest response to each border bar in A is ten spikes/second, then the highest response to each border bar in B could be, say, 15 spikes/second. Indeed, RT_B_ < RT_A_, as shown in Figure 5H. (Wolfson and Landy [44] observed a related phenomenon, more details in Li [22]). Furthermore, the highly salient vertical border bars make segmentation less susceptible to interference by task-irrelevant features, since their evoked responses are more likely dominating to dictate salience. Hence, interference in D is much weaker than in C, even though the task-irrelevant orientation contrast is 90° in both C and D. Indeed, RT_D_ < RT_C_ (Figure 5H), although RT_D_ is still significantly longer than RT_B_ without interference. All these are not due to any special status of the vertical orientation of the border bars in B and D, for rotating the whole stimulus patterns would not eliminate the effects. Similarly, when the task-irrelevant bars are uniformly oriented, as in patterns E and G (for A) and F (for B), the border in F is more salient than those in E and G, as confirmed by RT_F_ < RT_E_ and RT_G_.

The “protruding through” of the vertical border bars in D likely triggers the sensation of the (task-irrelevant) oblique bars as grouped or belonging to a separate (transparent) surface. This sensation arises more readily when viewing the stimulus in a leisurely manner rather than in the hurried manner of an RT task. Based on the arguments that one usually perceives the “what” after perceiving the “where” of visual inputs [45,46], we believe that this grouping arises from processes subsequent to the V1 saliency processing. Specifically, the highly salient vertical border bars are likely to define a boundary of a surface. Since the oblique bars are neither confined within the boundary nor occluded by the surface, they have to be inferred as belonging to another, overlaying (transparent), surface.

Given no orientation contrast between the task-irrelevant bars in E–G, the iso-orientation suppression among the irrelevant bars is much stronger than that in C and D, and is in fact comparable in strength to that among the task-relevant bars sufficiently away from the texture border. Hence, the responses to the task-relevant and -irrelevant bars are comparable in the background, and no interference would be predicted if we ignored general surround suppression between the relevant and irrelevant bars (detailed below). Indeed, RT_E_, RT_G_ ≪ RT_C_, and RT_F_ < RT_D_.

However, the existence of general surround suppression introduces a small degree of interference, making RT_E_, RT_G_ > RT_A_, and RT_F_ > RT_B_. Consider E for example, let us say that, without considering the general surround suppression, the relevant responses are ten spikes/second and five spikes/second at the border and background, respectively, and the irrelevant responses are five spikes/second everywhere. The general surround suppression enables nearby neurons to suppress each other regardless of their feature preferences. Hence, spatial variations in the relevant responses cause complementary spatial variations in the irrelevant responses (even though the irrelevant inputs are spatially homogeneous); see Figure 5I for a schematic illustration. For convenience, denote the relevant and irrelevant responses at the border as *O_border_*(*r*) and *O_border_*(*ir*) respectively, and as *O_near_*(*r*) and *O_near_*(*ir*)*,* respectively, at locations near but somewhat away from the border. The strongest general suppression is from *O_border_*(*r*) to *O_border_*(*ir*)*,* reducing *O_border_*(*ir*) to, say, four spikes/second. This reduction in turn causes a reduction of iso-orientation suppression on the irrelevant responses *O_near_*(*ir*)*,* thus increasing *O_near_*(*ir*) to, say, six spikes/second. The increase in *O_near_*(*ir*) is also partly due to a weaker general suppression from *O_near_*(*r*) (which is weaker than the relevant responses sufficiently away from the border because of the extra strong iso-orientation suppression from the very strong border responses *O_border_*(*r*) [47]). Mutual (iso-orientation) suppression between the irrelevant neurons is a positive feedback process that amplifies any response difference. Hence, the difference between *O_border_*(*ir*) and *O_near_*(*ir*) is amplified so that, say, *O_border_*(*ir*) = 3 and *O_near_*(*ir*) = 7 spikes/seconds, respectively. Therefore, *O_near_*(*ir*) dominates *O_near_*(*r)* somewhat away from the border, dictating and increasing the local saliency. As a result, the relative saliency of the border is reduced and some degree of interference arises, causing RT_E_ > RT_A_. The same argument leads similarly to conclusions RT_G_ > RT_A_ and RT_F_ > RT_B_, as seen in our data (Figure 5H). If colinear facilitation is not considered, the degree of interference in E and G should be identical, predicting RT_E_ = RT_G_. As explained below, considering colinear facilitation additionally will predict RT_E_ < RT_G_, as seen in our data for three out of four subjects (Figure 5H). Stimuli E and G differ in the direction of the colinear facilitation between the irrelevant bars. The direction is across the border in E but along the border in G, and, unlike iso-orientation suppression, facilitation tends to equalize responses *O_near_(ir)* and *O_border_(ir)* to the colinear bars. This reduces the spatial variation of the irrelevant responses across the border in E such that, say*, O_border_(ir)* = 4 and *O_near_(ir)* = 6 spikes/second, thus reducing the interference.

The SUM rule (over V1′s neural responses) would predict qualitatively the same directions of RT variations between conditions in this section only when the texture border highlight *H_border_* is measured by the ratio rather than by the difference between the (summed) response to the border and that to the background. However, using the same argument as in the section Interference by Task-Irrelevant Features, our quantitative data would make the SUM rule even more implausible than it is in that section (since, using the notations from that section, we note that *O_ground_* approximates the irrelevant responses in E and G, whose weak interference would require a constraint of *H_border_* = (*O_border_* + *O_ground_*)/2*O_ground_* > 1 + *δ* with *δ* ≫ 0, in addition to the other stringent constraints in that section that made the SUM rule less plausible).

We also carried out experiments in visual search tasks analogous to those in Figures 3–5, as we did in Figure 1E analogous to Figure 1D. Qualitatively the same results as those in Figures 3 and 4 were found; see Table 1. For visual search conditions corresponding to those in Figure 5, however, since there were no elongated texture borders in the stimuli, grouping effects arising from the colinear border, or as the result of the elongated texture border, are not predicted, and indeed, not reflected in the data; see Table 2. This confirmed additionally that saliency is sensitive to spatial configurations of input items in the manner prescribed by V1 mechanisms.

**Table 2:**
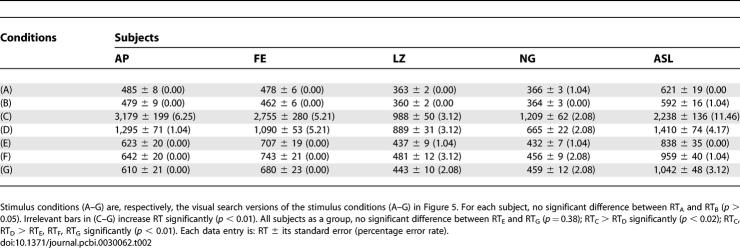
RTs (ms) for Visual Search for Unique Orientation, Corresponding to Data in Figure 5H

## Discussion

In summary, we tested and confirmed several predictions from the hypothesis of a bottom-up saliency map in V1. All these predictions are explicit since they rely on the known V1 mechanisms and an explicit assumption of a MAX rule, 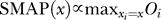 ; i.e., among all responses *O_i_* to a location *x,* only the most active V1 cell responding to this location determines its saliency. In particular, the predicted interference by task-irrelevant features and the lack of saliency advantage for orientation–orientation double features are specific to this hypothesis since they arise from the MAX rule. The predictions of color-orientation asymmetry in interference, the violation in the RT for color-orientation double feature of a race model between color and orientation features, the increased interference by larger color patches, and the grouping by spatial configurations, stem one way or another from specific V1 mechanisms. Hence, our experiments provided direct behavioral test and support of the hypothesis.

As mentioned in the Interference by Task-Irrelevant Features, the predicted and observed interference by irrelevant features, particularly those in Figures 1 and 2, cannot be explained by any background “noise” introduced by the irrelevant features [19,35], since the irrelevant features in our stimuli have a spatially regular configuration and thus would by themselves evoke a spatially uniform or non-noisy response.

The V1 saliency hypothesis does not specify which cortical areas read out the saliency map. A likely candidate is the superior colliculus which receives input from V1 and directs eye movements [48]. Indeed, microstimulation of V1 makes monkeys saccade to the RF location of the stimulated cell [26], and such saccades are believed to be mediated by the superior colliculus.

While our experiments support the V1 saliency hypothesis, the hypothesis itself does not exclude the possibility that other visual areas contribute additionally to the computation of bottom-up saliency. Indeed, the superior colliculus receives inputs also from other visual areas [48]. For instance, Lee et al. [49] showed that pop-out of an item due to its unique lighting direction is associated more with higher neural activities in V2 than those in V1. It is not inconceivable that V1′s contribution to bottom-up saliency is mainly for the time duration immediately after exposure to the visual inputs. With a longer latency, especially for inputs when V1 signals alone are too equivocal to select the salient winner within that time duration, it is likely that the contribution from higher visual areas will increase. This is a question that can be answered empirically through additional experiments (e.g., [50]) beyond the scope of this paper. These contributions from higher visual areas to bottom-up saliency are in addition to the top-down selection mechanisms that further involve mostly higher visual areas [51–53]. The feature-blind nature of the bottom-up V1 selection also does not prevent top-down selection and attentional processing from being feature selective [18,54,55], so that, for example, the texture border in Figure 1C could be located through feature scrutiny or recognition rather than saliency.

It is notable that while we assume that our RT data are adequate to test bottom-up saliency mechanisms, our stimuli remained displayed until the subjects responded by button press, i.e., for a duration longer than the time necessary for neural signals to propagate to higher level brain areas and feedback to V1. Although physiological observations [56] indicate that preparation for motor responses contribute a long latency and variations in RTs, our work needs to be followed up in the future to further validate our hopeful assumption that our RT data sufficiently manifest bottom-up saliency to be adequate for our purpose. We argue that to probe the bottom-up processing behaviorally, requiring subjects to respond to a visual stimulus (which stays on before the response) as soon as possible, is one of the most suitable methods. We believe that this method should be more suitable than an alternative method to present stimulus briefly, with, or especially without, requiring the subjects to respond as soon as possible. After all, turning off the visual display does not prevent the neural signals evoked by the turned-off display from being propagated to and processed by higher visual areas [57], and, if anything, it reduces the weight of stimulus-driven or bottom-up activities relative to the internal brain activities. Indeed, it is not uncommon for subjects to experience in RT tasks that they could not cancel their erroneous responses in time even though the error was realized way before the response completion and at the initiation of the response according to EEG data [58], suggesting that the commands for the responses were issued considerably before the completion of the responses.

Traditionally, there have been other frameworks for visual saliency [18,19,30], mainly motivated by and developed from behavioral data [4,5] when there was less knowledge of their physiological basis. Focusing on their bottom-up aspect, these frameworks can be paraphrased as follows. Visual inputs are analyzed by separate feature maps, e.g., red feature map, green feature map, vertical, horizontal, left-tilt, and right-tilt feature maps, etc., in several basic feature dimensions such as orientation, color, and motion direction. The activation of each input feature in its feature map decreases roughly with the number of the neighboring input items sharing the same feature. Hence, in an image of a vertical bar among horizontal bars, the vertical bar evokes a higher activation in the vertical feature map than that by each of the many horizontal bars in the horizontal map. The activations in separate feature maps are summed to produce a master saliency map. Accordingly, the vertical bar produces the highest activation at its location in this master map and attracts visual selection. The traditional theories have been subsequently made more explicit and implemented by computer algorithms [31]. When applied to the stimulus in Figure 1C, it becomes clear that the traditional theories correspond to the SUM rule 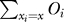 for saliency determination when different responses *O_i_* to different orientations at the same location *x* represent responses from different feature maps. As argued, our data (in the sections Interference from Task-Irrelevant Features, The Color Orientation Asymmetry in Interference, and Emergent Grouping of Orientation Features by Spatial Configurations) on interference by task-irrelevant features are incompatible with or unfavorable for the SUM rule, and our data (in the section Advantage for Color-Orientation Double Feature but Not Orientation–Orientation Double Feature) on the lack of advantage for the double-orientation contrast are contrary to the SUM rule. Many of our predictions from the V1 saliency hypothesis, such as the color-orientation asymmetry in the section The Color Orientation Asymmetry in Interference and the section Advantage for Color-Orientation Double Feature but Not Orientation–Orientation Double Feature, and the emergent grouping phenomenon in the section Emergent Grouping of Orientation Features by Spatial Configuration arise specifically from V1 mechanisms, and could not be predicted by traditional frameworks without adding additional mechanisms or parameters. The traditional framework also contrasted with the V1 saliency hypothesis by implying that the saliency map should be in higher-level cortical areas where neurons are untuned to features, motivating physiological experiments searching for saliency correlates in areas such as the lateral intraparietal area which, downstream from V1, could reflect bottom-up saliences in its neural activities [59,60]. Nevertheless, the traditional frameworks have provided an overall characterization of previous behavioral data on bottom-up saliency. These behavioral data provided part of the basis on which the V1 theory of saliency was previously developed and tested by computational modeling [20–23].

One may seek alternative explanations for our observations predicted by the V1 saliency hypothesis. For instance, to explain interference in Figure 1C, one may assign a new feature type to “two bars crossing each other at 45°,” so that each texture element has a feature value (orientation) of this new feature type. Then, each texture region in Figure 1C is a checkerboard pattern of two different feature values of this feature type. So the segmentation could be more difficult in Figure 1C, just like it could be more difficult to segment a texture of “ABABAB” from another of “CDCDCD” in a stimulus pattern “ABABABABABCDCDCDCDCD” than to segment “AAA” from “CCC” in “AAAAAACCCCCC.” This approach of creating new feature types to explain hitherto unexplained data could of course be extended to accommodate other new data. So for instance, new stimuli can easily be made such that new feature types may have to include other double feature conjunctions (e.g., color-orientation conjunction), triple, quadruple, and other multiple feature conjunctions, or even complex stimuli like faces, and it is not clear how long this list of new feature types needs to be. Meanwhile, the V1 saliency hypothesis is a more parsimonious account since it is sufficient to explain all the data in our experiments without evoking additional free parameters or mechanisms. It was also used to explain visual searches for, e.g., a cross among bars or an ellipse among circles without any detectors for crosses or circles/ellipses [20,23]. Hence, we aim to explain the most data by the fewest necessary assumptions or parameters. Additionally, the V1 saliency hypothesis is a neurally based account. When additional data reveal the limitation of V1 for bottom-up saliency, searches for additional mechanisms for bottom-up saliency can be guided by following the neural basis suggested by the visual pathways and the cortical circuit in the brain [48].

Computationally, bottom-up visual saliency serves to guide visual selection or attention to a spatial location to give further processing of the input at that location. Therefore, by nature of its definition, bottom-up visual saliency is computed before the input objects are identified, recognized, or decoded from the population of (V1) neural responses to various primitive features and their combinations. More explicitly, recognition or decoding from (V1) responses requires knowing *both* the response levels *and* the preferred features of the responding neurons, while saliency computation requires only the former. Hence, saliency computation is less sophisticated than object identification; it can thus be achieved more quickly (this is consistent with previous observations and arguments that segmenting or knowing “where is the input” is before or faster than classifying “what is the input” [45,46]), as well as more easily impaired or susceptible to noise. On the one hand, the noise susceptibility can be seen as a weakness or a price paid for a faster computation; on the other, a more complete computation at the bottom-up selection level would render the subsequent, attentive, processing more redundant. This is particularly relevant when considering whether the MAX rule or the SUM rule, or some other rule (such as a response power summation rule) in between these two extremes, is more suitable for saliency computation. The MAX rule to guide selection can be easily implemented in a fast and feature-blind manner, in which a saliency map readout area (e.g., the superior colliculus) can simply treat the neural responses in V1 as values in a universal currency bidding for visual selection, to select (stochastically or deterministically) the RF location of the highest bidding neuron [34]. The SUM rule, or for the same reason the intermediate rule, is much more complicated to implement. The RFs of many (V1) neurons covering a given location are typically non-identically shaped and/or sized, and many are only partially overlapping. It would be nontrivial to compute how to sum the responses from these neurons, whether to sum them linearly or nonlinearly, and whether to sum them with equal or non-equal weights of which values. More importantly, we should realize that these responses should not be assumed as being evoked by the same visual object—imagine an image location around a green leaf floating on a golden pond above an underlying dark fish—deciding whether and how to sum the response of a green tuned cell and that of a vertical tuned cell (which could be responding to the water ripple, the leaf, or the fish) would likely require assigning the green feature and the vertical feature to their respective owner objects, i.e., to solve the feature-binding problem. A good solution to this assignment or summation problem would be close to solving the object-identification problem, making the subsequent attentive processing, after selection by saliency, redundant. These computational considerations against the SUM rule are also in line with the finding that statistical properties of natural scenes also favor the MAX rule [61]. While our psychophysical data also favor the MAX over the SUM rule, it is currently difficult to test conclusively whether our data could be better explained by an intermediate rule. This is because, with the saliency map SMAP, RT = *f*(SMAP, ***β***) (see Equation 4) depend on decision making and motor response processes parameterized by ***β***. Let us say that, given V1 responses ***O*** *,* the saliency map is, generalizing from Equation 3, SMAP = SMAP(***O***, γ), where γ is a parameter indicating whether SMAP is made by the MAX rule or its softer version as an intermediate between MAX and SUM. Then, without precise (quantitative) details of ***O*** and **β**, γ cannot be quantitatively determined. Nevertheless, our data in Figure 4H favor a MAX rather than an intermediate rule for the following reasons. The response level to each background texture bar in Figure 4E–4G is roughly the same among the three stimulus conditions, regardless of whether the bar is relevant or irrelevant, since each bar experiences roughly the same level of iso-orientation suppression. Meanwhile, let the relevant and irrelevant responses to the border bars be *O_E_*(*r*) and *O_E_*(*ir*)*,* respectively, for Figure 4E, and *O_F_*(*r*) and *O_F_*(*ir*)*,* respectively, for Figure 4F. Then the responses to the two sets of border bars in Figure 4G are approximately *O_E_*(*r*) and *O_F_*(*r*), ignoring, as an approximation, the effect of increased level of general surround suppression due to an increased level of local neural activities. Since both *O_E_*(*r*) and *O_F_*(*r*) are larger than both *O_E_*(*ir*) and *O_F_*(*ir*), an intermediate rule (unlike the MAX rule) combining the responses to two border bars would yield a higher saliency for the border in Figure 4G than for those in Figure 4E and Figure 4F, contrary to our data. This argument, however, cannot conclusively reject the intermediate rule, especially one that closely resembles the MAX rule, since our approximation to omit the effect of the change in general surround suppression may not hold.

Due to the difference between the computation for saliency and that for discrimination, it is not possible to predict discrimination performance from visual saliency. In particular, visual saliency computation could not predict subjects' sensitivities, e.g., their d prime values, to discriminate between two texture regions (or to discriminate the texture border from the background). In our stimuli, the differences between texture elements in different texture regions are far above the discrimination threshold with or without task-irrelevant features. Thus, if instead of an RT task, subjects performed texture discrimination without time pressure in their responses, their performance will not be sensitive to the presence of the irrelevant features (even for briefly presented stimuli) since the task essentially probes the visual process for discrimination rather than saliency. Therefore, our experiments to measure RT in a visual segmentation or search task, requiring subjects to respond quickly regarding “where” rather than “what” about the visual input by pressing a button located congruently with “where,” using trivially discriminable stimuli, are designed to probe bottom-up saliency rather than the subsequent object recognition (identification) or discrimination performance. This design assumes that a higher saliency of the texture border or the search target makes its selection easier and thus faster, manifesting in a shorter RT. This is why our findings in RTs cannot be explained by models of texture discrimination (e.g., [62]), which are based on discriminating or identifying texture features, i.e., based on visual processing after visual selection by saliency. While our subjects gave different RTs to different stimuli, their response error rates are typically very small (<5%) to all stimuli—as our RT task is not to measure discrimination sensitivities (or d prime values). For the same reason, if one were to explain the interference in Figure 1C by the noise added by the task-irrelevant features, this feature noise would not be strong enough to sufficiently affect the error rate, since the feature differences (between those of the irrelevant and relevant features) are many times larger than the just-noticeable feature difference for feature discrimination. Of course, some visual search tasks, especially those using hardly discriminable stimuli, rely more on the recognition and/or less on bottom-up saliency computation. These tasks, while interesting to study for other purposes, would not be suitable for testing hypotheses on the bottom-up saliency, and we expect that cortical areas beyond V1 would be more involved for them and would have to read out from V1 the preferred features (labeled lines) *and* activities of more *and* less active neurons (i.e., beyond reading out the SMAP).

Our observations are related to Gestalt principles of perceptual organization and many previous observations of visual grouping and emergent properties [63,64]. This suggests that V1 mechanisms could be the neural basis for many grouping phenomena, as has been shown in some examples [47,65]. For instance, the main Gestalt principle of grouping by similarity is related to iso-feature suppression in V1, since iso-feature suppression, responsible for feature singleton pop-out, also makes a region of items of similar features less salient apart from the region border, which bounds, and induces the perception of, the region as a whole. Similarly, the principle of grouping by proximity is related to the finite length of the intracortical connections in V1 for contextual influences, and the principle of grouping by good continuation is related to the colinear facilitation in V1. Pomerantz [63] showed that certain features, particularly ones involving spatial properties such as orientation, interact in complex ways to produce emergent perceptual configurations that are not simply the sum of parts. One of his notable examples of what is termed “configuration superiority effect” is shown in Figure 6. One stimulus of a left-tilted bar among three right-tilted bars becomes a composite stimulus of a triangle among three arrows, when a non-informative stimulus of four identical “L”-shaped items is added. As a result, the triangle is easier to detect among the arrows than the left-tilted bar among right-tilt ones in the original stimulus, as if the triangle is an emergent new feature. This superiority effect by spatial configurations of bars, the opposite of interference by irrelevant features in our data, could be accounted for by the following mechanism beyond V1. The added irrelevant “L”s made the target triangle shape unique, while the original target bar was a rotated version of the bar distractors. It was recently shown [66] that, when the bottom-up saliency is not sufficiently high (as manifested in the longer-than-1,000-ms RTs in Pomerantz's data, likely due to a small set size), object rotational invariance between target and distractors could introduce object-to-feature interference to drastically prolong RT. This interference is because the original target, identically shaped as distractors, is confused as a distractor object. Whereas Gestalt principles and many psychological studies of emergent phenomena have provided excellent summaries and descriptions of a wealth of data, the V1 mechanisms provide explanations behind at least some of these data.

**Figure 6:**
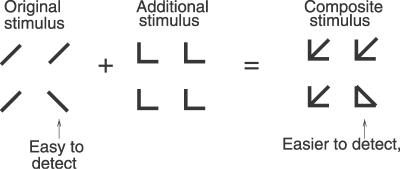
Illustration of Pomerantz's Configuration Superiority Effect The triangle is easier to detect among the three arrow shapes in the composite stimulus, than the left-tilted bar among the right-tilted bars in the original stimulus. Identical shape of the target and distractor bars in the original stimulus could lead to confusion and longer RT.

Meanwhile, the psychological data in the literature, including the vast wealth of data on visual grouping, can in turn predict the physiology and anatomy of V1 through the V1 saliency hypothesis, thus providing opportunities to further test the hypothesis through physiological/anatomical experiments. Such tests should help to explore the potentials and the limitations of the V1 mechanisms to explain the bottom-up selection factors. For example, knowing that color-orientation conjunctive search is difficult (e.g., [37], searching for a red vertical target among red horizontal and green vertical distractors) and that color-orientation double feature is advantageous allow us to predict that, in V1, intracortical (disynaptic) suppressive connections should link conjunctive cells with other cells preferring either the same color and/or the same orientation. Data by Hegde and Felleman [28] are consistent with this prediction, although more direct and systematic tests of the prediction are desirable. Similarly, the ease to search for a unique motion–orientation (or motion–form) conjunction predicts [23] that V1 cells tuned to motion–orientation conjunctions tend to connect to other cells preferring both the same orientation and the same motion direction.

The V1 mechanisms for bottom-up saliency also have implications for mechanisms of top-down attention. First, if V1 creates a bottom-up saliency map for visual selection, then it would not be surprising that subsequent cortical areas/stages receiving input from V1 should manifest much interaction between bottom-up and top-down selectional and attentional factors. Second, by the V1 saliency hypothesis, the most active V1 cell attracts attention automatically to its RF location. This cell may be tuned to one or a few feature dimensions. Its response does not provide information about other feature dimensions to which it is un-tuned. Thus, such a bottom-up selection does not bind different features at the same location, and the top-down attention may have to bind the features subsequently [4]. Meanwhile, the conjunctive cells in V1 bind two (or more) features at the same location into a single cell by default (which may or may not be veridical). This suggests that top-down attentional mechanisms are required to determine, from the responses of the conjunctive and nonconjunctive cells, not only the relative strengths of the two features, but also whether the two features belong to the same objects or whether the two features need to be unbound. Our findings reported here should motivate researchers in new directions for research into the mechanisms and frameworks of bottom-up and top-down attentional selection, and post-selectional processes for problems including feature binding.

## Materials and Methods

### Stimuli.

In all our experiments, each stimulus pattern had 22 rows × 30 columns of items (of single or double bars) on a regular grid with unit distance 1.6° of visual angle. Each bar was a white (CIE illuminant C), 1.2 × 0.12 degree rectangle (for experiments in orientation feature dimensions only), or a colored 1.2 × 0.24 degree rectangle (for experiments involving color and orientation features). All bars had a luminance of 14 cd/m^2^ unless otherwise stated, and the background was black. The colored bars were green or pink specified by their CIE 1976 coordinates (u′,v′), with hue angles *h_uv_* = 130° or 310°, respectively, where tan(*h_uv_*) = (*v*′ – *v_n_*′)/(*u*′ – *u_n_*′), and (*u_n_*′, *v_n_*′) are the coordinates of CIE illuminant C (0.201, 0.461). All bars within a stimulus had the same saturation

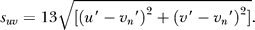
For segmentation experiments, the vertical texture border between two texture regions was located randomly left or right, at 7, 9, or 11 interelement distances laterally from the display centre. Stimuli in search tasks were made analogously to those in texture-segmentation tasks, by reducing one of the two texture regions into a single target item. In each trial, the target was positioned randomly in one of the middle 14 rows; given the target's row number, its column number was such that the target was positioned randomly left or right, as close as possible to 16.8° of visual angle from the display centre. The noncoloured bars are oriented either as specified in captions of the figures and tables presented, or are oriented horizontally, vertically, or ±45° from vertical. The color and orientation of the target or left texture region in each trial were randomly green or pink (for colored stimuli) and left- or right-tilted (or horizontal or vertical) in the relevant orientations.

### Subjects.

Subjects are adults with normal or corrected to normal vision, and they are identified by letters, such as LZ, in the figures and tables. Most subjects are naive to the purpose of the study, except for LZ (one of the authors), LJ, and ASL. Some subjects are more experienced at RT tasks than others. AP, FE, LZ, NG, and ASL participated in more experiments than others (such as KC, DY, and EW) who only participated in one or a few experiments.

### Procedure and data analysis.

Subjects were instructed to fixate centrally until stimulus onset, to freely move their eyes afterward, and to press a left or right key (located to their left or right hand side) using their left or right hand, respectively, quickly and accurately to indicate whether the target or texture border (present in each trial) was in the left or right half of the display. The stimulus pattern stayed after onset until the subject's response. There were 96 trials per subject per stimulus conditions shown. Average RTs were calculated (and shown in the figures and tables) excluding trials that were erroneous or had an RT outside three standard deviations from the mean. The number of such excluded trials was usually less than 5% of the total for each subject and condition, and our results did not change qualitatively even when we included all trials in calculating RTs or considered the speed–accuracy tradeoff in performances. The error bars shown are standard errors. The experiments were carried out in a dark room. Within each figure plot, and each part (A, B, C, etc.) of Table 1 or Table 2, all the stimulus conditions were randomly interleaved within an experimental session such that the subjects could not predict before each trial which stimulus condition would appear. For texture segmentation, the subjects were told to locate the border between two textures regardless of the difference (e.g., whether in color or orientation or both) between the two textures. For visual search, the subjects were told to locate the target which had a unique feature (such as orientation, color, or both, regardless of which orientation(s) and/or which color), i.e., the odd one out, within the display. The subjects were shown examples of the relevant stimulus conditions to understand the task before the data-taking. Experiments (e.g., the one for Figure 5) requiring more than 300–400 trials in total were broken down to multiple data–taking sessions such that each session typically took 10–20 minutes.

## Abbreviations

RF: receptive field
RT: reaction time
VI: primary visual cortex
